# Neuroscience and psychiatric patients: does the brain matter?

**DOI:** 10.3325/cmj.2013.54.598

**Published:** 2013-12

**Authors:** Lukasz M. Konopka

**Affiliations:** Department of Psychiatry, Loyola Medical Center, Maywood, IL, USA and Yellowbrick Consultation and Treatment Center, Evanston, IL, USA

The field of neuroscience includes a broad spectrum of activities, methods, and interests. Among other things, it investigates the very basic genetic contributions of phenotype, role of genes in health and disease, and how genetic factors help identify approproate medications based on the body’s ability to metabolize these chemicals (pharmacokinetic profiles). Neuroscience extends beyond the microscopic to how the brain responds to simple environmental stimuli such as visual, auditory, and somatosensory input and to the more complex processes of conversations, decision making, emotional experiences, rewards, punishments, pain, and pleasure. It also includes our understanding of molecular processes, the variation of synaptic transmission, resulting electrical changes across cellular membranes, and modification of intracellular processes through protein activation, deactivation, or modulation. Intracellular modulation resulting form external or internal input establishes new, modified synapses, which increase the neuronal network complexity and integration and result in observable behavioral expression. These processes are commonly referred to as brain plasticity (1).

We understand that one key element of plasticity is the modulation of brain derived growth factors (BDNF) (2). Ironically, not long ago, neuroscientists believed that the mature adult brain lacked the ability to respond to new challenges and that, outside of progressive deterioration, the brain was incapable of remodeling. This implied that past a certain age we were incapable of learning. We now know that this is not true. The healthy brain is capable of learning throughout life (3,4) and given the appropriate milieu it adapts to environmental challenges at both the cellular and behavioral levels (2). Unfortunately, even today in some academic settings we still talk about the brain process as behavioral or organic in search of possible “organicity.” This concept was acceptable in the past because we did not have evidence that behavioral symptoms were related to changes in brain physiology. Interestingly, the concept of “organicity” emerged with the development of basic imaging instruments, ie, pneomoencephalography, despite questionable quality of brain images (5). In some cases, it was, and still is, assumed that a negative, normal radiology report reflects absence of “organicity.” This is particularly true in psychiatric patients, especially when we use standard CT or MRI. When we receive a normal imaging report, the tendency is to suggest that the patient is dealing with purely psychological/psychiatric processes unrelated to the underlying neurobiology. Thus, we infer that behavior is independent of structure and function (6). This belief needs to be questioned. Structural CT and MRI data only begin to enhance our understanding of dysfunction. For a complete assessment of abnormality, we need to conduct functional studies that include blood perfusion and metabolism measures, oxygen utilization, receptor mapping, and electrophysiology. Without these measures, we will have incomplete diagnosis and, subsequently, inferior patient care (7).

There are significant inconsistencies in psychiatric patient care. For example, we inconsistently and haphazardly treat patients with psychotropic medications, which influence the brain and body by modifying the pharmacodynamics of neurotransmitter receptor interactions. These pharmacological interventions are utilized even by non specialists. For instance, pediatricians prescribe medications to treat attention deficit disorders in children without much consideration for the potential impact of these mediations on the developing brain (8). These approaches are common in spite of new developments in human genomics research that can identify individual pharmacokinetic (9) and some pharmacodynamic (10) vulnerabilities. This information is widely available but not used routinely in the clinical settings. As mentioned earlier, when we modify transmitter receptor relations, we may modulate gene expression and activate or inhibit protein synthesis. This is particularly true with medications whose mechanisms of action involve metabotropic receptor systems. Because metabotropic receptors dominate the brain, these medications are likely to have widespread and lasting effects on the physiology of the neurons and subsequently on their neuronal networks. Keeping this in mind we should think about the medication effects at the level of neurons as well as the level of behavior that we wish to modify. This requires our understanding of the brain’s normal and abnormal functions and assessment of these before, during, and after intervention. In the face of growing neuroscience data, we may postulate that all psychiatric conditions reflect aberrant brain physiology. Unfortunately, even today common psychiatric practice relies on categorizing patients based on symptoms reported by the patient and, occasionally, assessment obtained by self-report measures or clinician conducted interviews. The standards of psychiatric practice ignore the vast neuroscientific data indicating that there is a strong relationship between brain anatomy and physiology and correlating to specific presenting symptoms, as well as neuropsychological profiles that impact how patients view the internal and external world and how they respond to the content and quality of the information presented to them (11).
